# Assessing the perceived changes in neighborhood physical and social environments and how they are associated with Chinese internal migrants’ mental health

**DOI:** 10.1186/s12889-021-11289-4

**Published:** 2021-06-28

**Authors:** Min Yang, Julian Hagenauer, Martin Dijst, Marco Helbich

**Affiliations:** 1 Department of Human Geography and Spatial Planning, Faculty of Geosciences, Utrecht University, Princetonlaan 8A, Utrecht, CB 3584 The Netherlands; 2 LISER, Luxembourg Institute of Socio-Economic Research, Esch-sur-Alzette, Luxembourg; 3 University of Luxembourg, Esch-sur-Alzette, Luxembourg

**Keywords:** Migrants, Neighborhood changes, Moving trajectory, Relocation, Mental health, Machine learning

## Abstract

**Background:**

Migrants experience substantial changes in their neighborhood physical and social environments along their migration journeys, but little is known about how perceived changes in their neighborhood environment pre- and post-migration correlate with their mental health. Our aim was to examine the associations between recalled changes in the perceived neighborhood physical and social environments and migrants’ mental health in the host city.

**Methods:**

We used cross-sectional data on 591 migrants in Shenzhen, China. We assessed their risk of mental illness using the General Health Questionnaire (GHQ). Neighborhood perceptions were collected retrospectively pre- and post-migration. We used random forests to analyze possibly non-linear associations between GHQ scores and changes in the neighborhood environment, variable importance, and for exploratory analysis of variable interactions.

**Results:**

Perceived changes in neighborhood aesthetics, safety, and green space were non-linearly associated with migrants’ mental health: A decline in these characteristics was associated with poor mental health, while improvements in them were unrelated to mental health benefits. Variable importance showed that change in safety was the most influential neighborhood characteristic, although individual-level characteristics—such as self-reported physical health, personal income, and hukou (i.e., the Chinese household registration system)—appeared to be more important to explain GHQ scores and also strongly interacted with other variables. For physical health, we found different associations between changes in the neighborhood provoked by migration and mental health.

**Conclusion:**

Our findings suggest that perceived degradations in the physical environment are related to poorer post-migration mental health. In addition, it seems that perceived changes in the neighborhood environment play a minor role compared to individual-level characteristics, in particular migrants’ physical health condition. Replication of our findings in longitudinal settings is needed to exclude reverse causality.

**Supplementary Information:**

The online version contains supplementary material available at 10.1186/s12889-021-11289-4.

## Background

Between 1979 and 2019, China’s urban population increased from 17 to 60% [1]. The country’s annual economic burden caused by mental disorders quadrupled from $21.0 billion to $88.8 billion between 2005 and 2013 [2]. Internal migrants contributed significantly to China’s growing urban population, and they are particularly vulnerable to mental illness resulting from changes in their social and physical living environments [3–5].

Evidence is mounting that risk factors for mental illness in host cities are different for migrants compared to non-migrants; for example, migrants tend to have a low socioeconomic status, are separated from their families, and are socially excluded from their host societies [6–9]. Furthermore, hukou (i.e., the Chinese household registration system) prevents migrants from accessing social and medical benefits in host cities. Failure to transfer their hukous to host cities puts migrants at additional mental health risk because of the associated social welfare and healthcare inequalities [10].

Studies have shown that good neighborhood physical and social environments contribute to mental health among the general population [11]; in this, the social environment seems to play a more important role [12]. For example, noise [13], air pollution [14], lack of essential neighborhood facilities (e.g., lighting, benches) [15], and safety concerns [16] were found to threaten mental health. In contrast, neighborhood social cohesion and social support [17] may contribute to better mental health. The physical environment in the form of green space may also promote mental health [18].

Previous studies on neighborhood–mental health relationships were limited in two ways. First, it remains unclear whether such associations hold for migrants experiencing changes in their living environment due to moving. This concern echoes criticism put forward elsewhere [19] that neighborhood environments are measured at a single point in time [20–22], which may lead to over-emphasizing the role of the neighborhood in which people currently live. Only a few studies have incorporated neighborhood experiences at multiple stages of people’s lives [23–25]. It is plausible that migrants’ mental health is shaped by both the previous and the actual neighborhood environment [19, 26, 27]. For example, a US study showed that moving away from an impoverished neighborhood resulted in improved mental health [28].

Second, some studies (i.e., [29–31]) have a methodological deficit because they were mainly based on inflexible regression analyses assuming that neighborhood–mental health correlations are linear. While there is no plausible reason for such a simplification, this may at least partly contribute to inconsistent results across studies [32]. To overcome these constraints, others have promoted the application of more flexible machine learning approaches to uncover complex and possibly non-linear associations [12]. Non-linearities make intuitive sense, because people’s mental health might not respond in the same way to an improvement or deterioration of the neighborhood environment. Meanwhile, several machine learning models (e.g., random forests) have demonstrated their analytical advantages by routinely assessing the importance of explanatory variables and modeling variable interactions [33], which supports our understanding concerning the mechanisms underlying how neighborhood characteristics correlate with mental health.

To address these limitations, the present study used random forests to examine associations between the perceived changes in neighborhood physical and social environments pre- and post-migration and migrants’ mental health in the host city of Shenzhen, China. Three research questions were formulated:

1) How are recalled changes in perceived neighborhood characteristics associated with migrants’ mental health in the host city?

2) Which perceived neighborhood changes are most influential for migrants’ mental health?

3) How do migrants’ individual-level characteristics interact with neighborhood-level characteristics in associations with current mental health?

## Materials and methods

### Study area and study design

A cross-sectional observational study was conducted in the city of Shenzhen, China. Shenzhen has a population of 11.37 million people, 70% of whom are migrants [34], which made it an ideal site for our study.

Data were collected by means of a survey carried out between January and April 2017. We selected participants using a stratified sampling design. First, two inner-city districts (Nanshan and Futian) and two suburban districts (Longgang and Baoan) were chosen as sampling areas. Second, within each of the districts, five neighborhood types were identified based on the neighborhood physical environment and the socioeconomic composition of the residents. These neighborhood types can be broadly defined as work unit compound, inner-city village, commodity housing community (a private real estate development), social housing, and factory dormitory. Previous research stressed the differences in socioeconomic composition and physical environment between different neighborhood types [35]. Finally, we took a random sample of people per selected neighborhood. Eligible to participate were people older than 18 years who had lived in Shenzhen for at least 6 months. In total, our sample included 855 respondents. After removing non-migrants (i.e., people born in Shenzhen, *N* = 264), the final sample comprised 591 people.

### Data

#### Mental health as outcome variable

Migrant’s self-reported mental health was assessed using the well-tested Chinese version [36] of the 12-item General Health Questionnaire (GHQ-12) [37]. The GHQ-12 is a psychometric screener dealing with people’s emotions and daily functioning in the 4 weeks prior to the survey. The items address respondents’ experience of self-confidence, losing sleep, and other psychiatric conditions. Each item was scored on a 4-point Likert scale ranging from 0 (not at all) to 3 (yes, always). The 12 individual scores were summed, leading to a total score that served as our outcome variable. The GHQ-12 in our sample ranged from 0 (good mental health) to 30 (poor mental health). A Cronbach’s alpha of 0.821 indicated the good internal consistency of the GHQ-12 in our sample.

#### Perceptions of the residential neighborhood pre- and post-migration

We assessed the physical and the social dimension of migrants’ residential neighborhoods pre- and post-migration. Although longitudinal data are preferred to measure neighborhood perceptions, our retrospective approach captures migrants’ neighborhood experiences with minor recall bias [38, 39].

The neighborhoods’ physical characteristics were measured by means of the short version of the Neighborhood Environment Walkability Scale (NEWS-A) [40]. We used 13 of the 54 NEWS-A items to represent neighborhood facilities and their accessibility (4 items), aesthetics (4 items), safety (2 items), and the availability of green space (3 items). Respondents were asked to assess their neighborhoods by means of a 5-point Likert scale. The items for each attribute were then summed, with higher scores indicating better perceived neighborhood characteristics.

The neighborhood social environment was assessed by social cohesion, which was operationalized by asking respondents to rate the following statements on a 5-point Likert scale ranging from 1 (totally disagree) to 5 (totally agree): “People in the neighborhood are willing to help their neighbors”, “The neighborhood organization is very helpful”, “People in the neighborhood share the same values”, “The neighborhood is close-knit”, “People in the neighborhood can be trusted” [41]. The scores were summed, with higher scores indicating better perceived neighborhood social environments.

Changes in the perceived neighborhood environment were operationalized by subtracting the scores of the previous neighborhood from those of the current neighborhood. Values below 0 indicate a decline in a specific neighborhood attribute post-migration, while values above 0 indicate an improvement.

#### Individual-level variables

Following previous studies [10, 42] the following personal characteristics were included: age (measured in years), sex (male, female), education (high school or lower, bachelor’s degree, master’s or higher degree), personal monthly income (in CNY/month [1 CNY = 0.14 USD]), employment status (employed, unemployed), hukou type (a dummy variable referring to holding or not holding a Shenzhen hukou), and a variable representing the migration context (intra- or inter-province migration). We also considered self-rated physical health, as earlier studies suggested a relation between physical and mental health [10, 43]. Physical health was measured on a 5-point scale from poor to excellent [44].

### Machine learning-based analyses

Summary statistics were used to describe the study population. While there are numerous machine learning algorithms, studies have shown that random forests are competitive in their performance compared to other state-of-the-art machine learning models [12, 45]. For example, a competition of 38 algorithms concluded that the random forest model [33] has an outstanding predictive performance on a moderately large dataset and is a good initial algorithm choice [46].

We applied a random forest model [33, 47] to assess the associations between GHQ-12 scores and environmental changes between migrants’ previous and current neighborhoods. A random forest has the advantage that it models non-linear associations, includes interactions between variables, is not grounded on statistical assumptions, and is robust against overfitting [48]. Briefly, a random forest is a regression method that consists of a large number of different trees (i.e., an ensemble of trees) [33]. Each tree is built based on a bootstrap sample (i.e., sampling with replacement) of the data. When growing an individual tree, at each node only a random subset of the independent variables is selected which reduces the correlations among the trees. Finally, trees are grown to maximum size without any pruning and the overall predictions of the random forest are obtained by averaging the predictions from the individual regression trees.

Four approaches were used to obtain an in-depth model understanding. First, we assessed the variable importance by measuring how much, on average, each variable decreases the variance when the trees are grown (see, e.g., [49] for details). Second, we used partial dependence plots—which show the change in the average predicted value as one or more variables vary over their marginal distribution [50]—to investigate the directions and shape of the associations. Third, we quantified the total interaction of one variable with the other variables by means of the H statistic [51]. The statistic measures the variance of the difference between the observed partial dependence function and the decomposed one without interactions [52]. It can be calculated to evaluate the interaction of two variables or the overall interaction of one variable with all other variables. Values close to zero indicate no variable interactions, while values close to 1 indicate that the entire variance is explained by the partial dependence functions. Fourth, we visualized the interactions of selected variables (i.e., those having a high H statistic) using bivariate partial dependence plots. The analyses were carried out in R software [53] using the ranger package [54].

## Results

### Descriptive statistics

Table 1 presents the descriptive statistics of the study population. Our sample had a mean (μ) GHQ-12 score of 6.610 with a standard deviation (SD) of ±4.070. The average age of our respondents was 31.374 years (SD ± 7.911) and 44% were female. Both age and sex distributions of our sample matched closely the demographic profile of people in Shenzhen in 2018, when the mean age of the permanent residents was 32.5 years and 46% of the permanent residents were female (Statistics Bureau of Shenzhen Municipality, 2019). Because only a few respondents reported “poor” physical health, we merged the “poor” and the “fair” group into a “fair/poor” group, which accounted for 35% of the sample. Well over half (66%) of the respondents had at least good physical health. Over two thirds (69%) of the respondents were currently living in Shenzhen without a Shenzhen hukou, and 68% of these respondents had migrated from places outside the province (e.g., Guangdong). The average length of residence in Shenzhen was 4.350 years (SD ± 4.398). Approximately 34% of respondents lived in Shenzhen for 1 year or less.

**Table 1:** Descriptive statistics of the study population

Variable	Category	Minimum	Maximum	Mean	SD	Percentage
GHQ-12 scores		0.000	30.000	6.613	4.070	
*Changes in neighborhood characteristics*						
Aesthetics		−16.000	20.000	−0.132	4.073	
Safety		−8.000	8.000	−0.029	1.855	
Facilities and accessibility		−16.000	16.000	0.174	3.986	
Green space		−12.000	12.000	0.240	3.092	
Social cohesion		−20.000	20.000	−0.052	4.681	
*Individual-level characteristics*						
Age		17.000	68.000	31.374	7.911	
Physical health	Fair/poor					35%
	Good					31%
	Very good					22%
	Excellent					13%
Hukou type	Non-Shenzhen					69%
	Shenzhen					31%
Migration context	Intra-province					32%
	Inter-province					68%
Sex	Female					44%
	Male					56%
Education	High school or lower					32%
	Bachelor’s					62%
	Master’s and higher					6%
Income	≤4000 CNY					23%
	4001–8000 CNY					43%
	> 8000 CNY					34%
Employment	Unemployed					23%
	Employed					77%

Table 2 shows the changes in the perceived neighborhood environment pre- and post-migration. Approximately one third of the respondents reported improvements in the neighborhood environment, one third remained stable, and one third experienced a decline after moving to Shenzhen. The perceived changes in neighborhood conditions showed that, on average, the respondents experienced a slight decline in neighborhood quality post-migration in terms of aesthetics μ= − 0.132, SD ± 4.070), safety (μ= − 0.029, SD ± 1.866), and social cohesion (μ= − 0.052, SD ± 4.681). The neighborhood facilities and accessibility (μ=0.174, SD ± 3.986) and green space (μ=0.240, SD ± 3.092) improved post-migration.

**Table 2:** Changes in the perceived neighborhood environment pre- and post-migration

	Decline	Stable	Improvement
Perceived changes in neighborhood aesthetics	38%	24%	38%
Perceived changes in neighborhood safety	33%	38%	29%
Perceived changes in facilities and accessibility	35%	30%	35%
Perceived changes in neighborhood green space	32%	31%	37%
Perceived changes in the neighborhood social environment	36%	26%	38%

### Variable importance

**Fig. 1:**
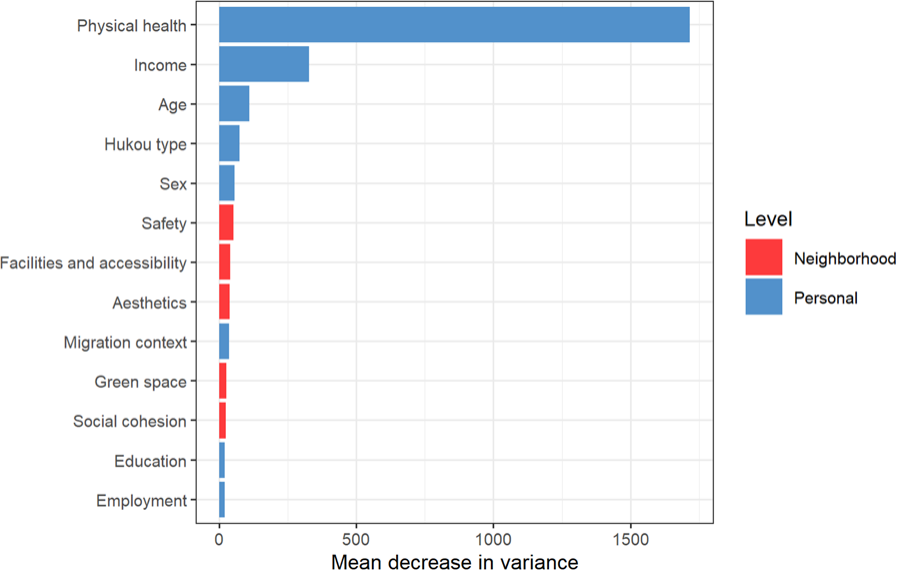
Variable importance of the individual-level and neighborhood-level characteristics. The higher the mean decrease in variance, the more important the variable

Figure 1 shows the variable importance for explaining migrants’ GHQ-12 scores. Physical health, income, and age were found to be the most important variables correlating with GHQ-12, followed by migrants’ hukou type and sex. Perceived changes in neighborhood characteristics were less important in explaining migrants’ mental health in the host city. The most irrelevant variables were education and employment status.

### Correlation analysis

**Fig. 2:**
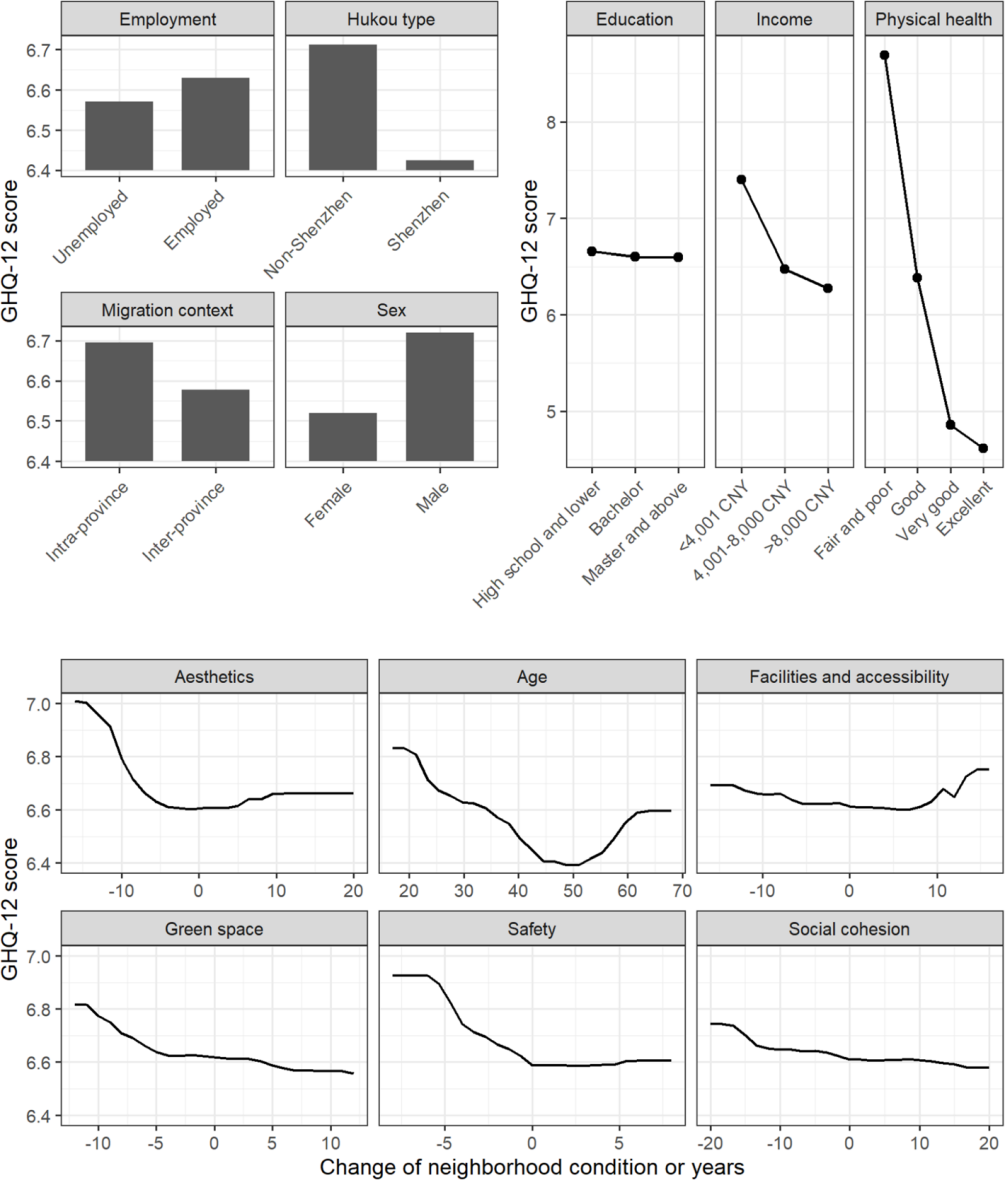
Partial dependence plots relating each independent variable to the GHQ-12 scores

Figure 2 shows the correlations between the GHQ-12 scores and the independent variables as partial dependence plots. A decrease in a migrant’s perception of safety was associated with a higher GHQ-12 score (meaning poorer mental health), while an increase was not associated with a lower GHQ-12 score (meaning better mental health). A similar pattern was found for perceived changes in neighborhood green space and aesthetics, where a reduction in the experiences of green space and aesthetics were associated with higher GHQ-12 scores. A decrease in GHQ-12 scores was observed for respondents with improvements (above 5) in green space. A decline in the perceived social cohesion post-migration was associated with higher GHQ-12 scores, and an increase with lower GHQ-12 scores.

As for individual-level characteristics, migrants with a Shenzhen hukou had lower GHQ-12 scores than those without a Shenzhen hukou. Inter-province migrants also had better mental health than intra-province migrants. Male migrants had higher GHQ-12 scores than female migrants. The overall trend indicated that better self-rated physical health was associated with lower GHQ-12 scores. There was a substantial drop in GHQ-12 scores between migrants who assessed their physical health as “fair/poor” and migrants who reported “very good” physical health. In contrast, the GHQ-12 difference between “very good” and “excellent” physical health was minor. A similar trend was found for income, whereby higher income levels were associated with lower GHQ-12 scores; the GHQ-12 score difference between the low- and the middle-income group was greater than that between the middle- and the high-income group.

### Variable interactions

**Fig. 3:**
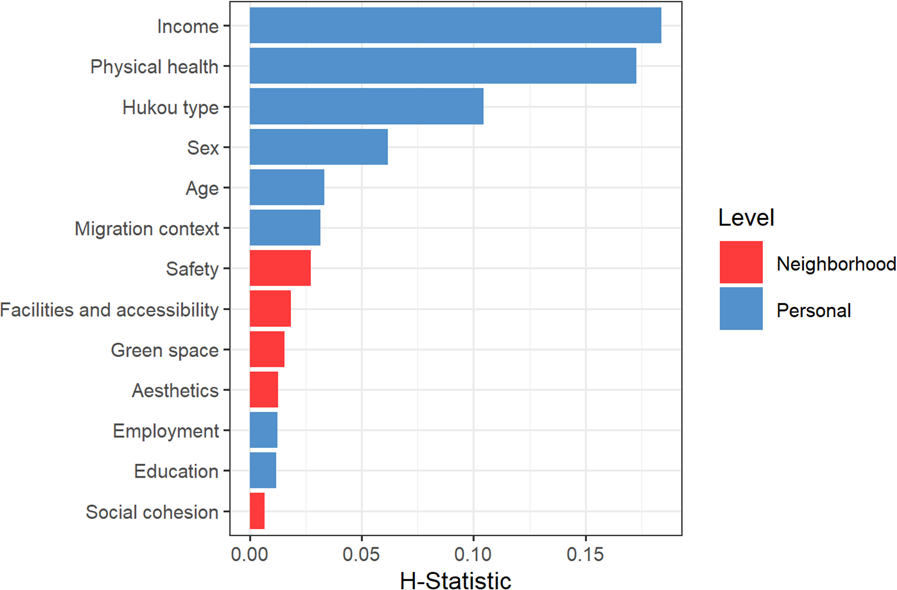
Overall variable interactions of the individual-level and changes in neighborhood characteristics. The higher the H statistic, the stronger a variable’s interaction with the other variables

**Fig. 4:**
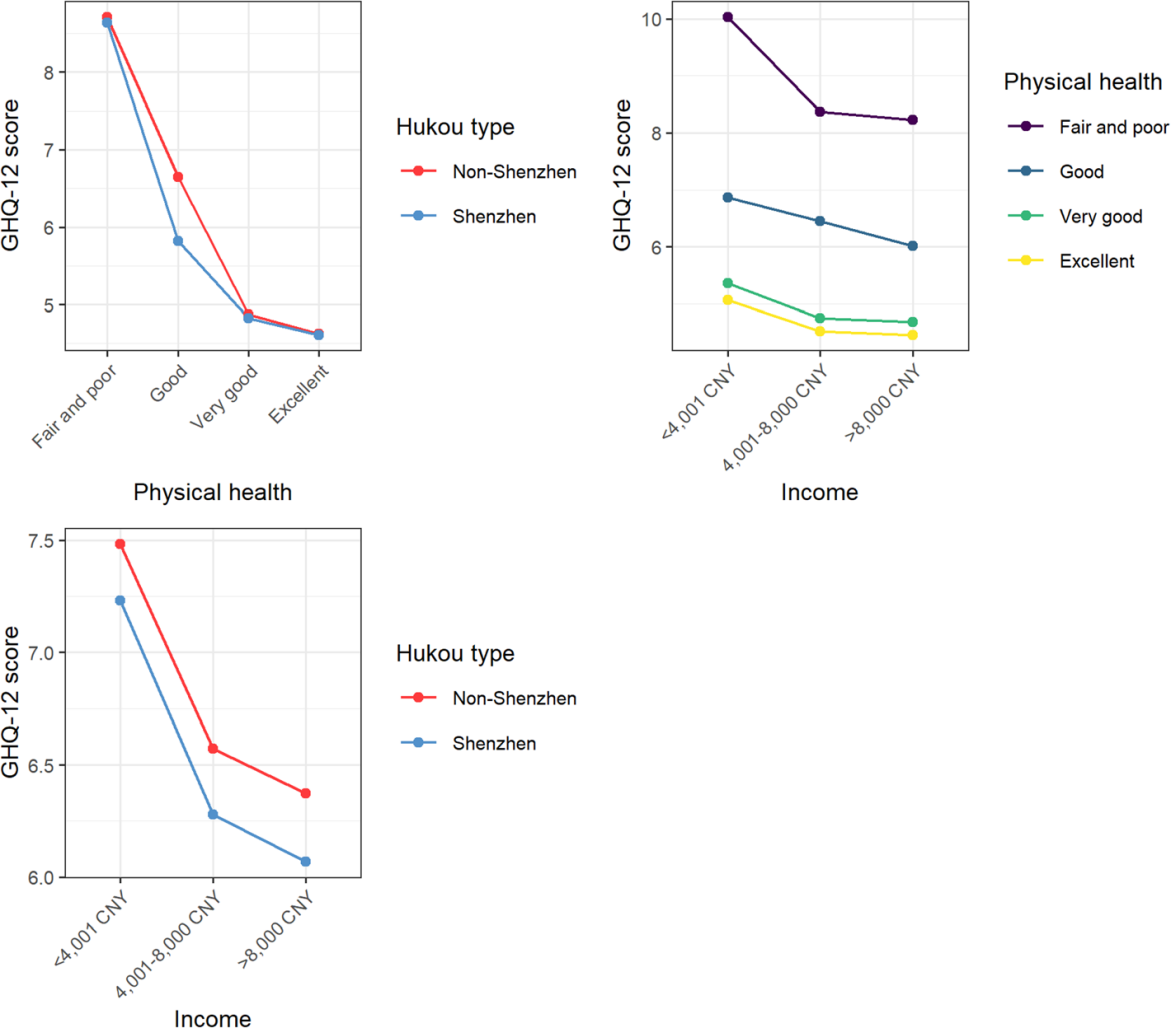
Bivariate partial dependence plots of income, hukou, and physical health

We further explored the variable interactions, Fig. 3 shows the H statistics for the variables used to evaluate variable interactions. Physical health, income, hukou type, and sex had pronounced overall interaction. The remaining variables, including all neighborhood characteristics, showed moderate to little interaction. To investigate the most important interactions among variables, we calculated bivariate partial dependence plots for those with high overall interaction (i.e., physical health, income, hukou type, and sex) (Fig. 4). Less substantial bi-variate interactions are provided in the supplementary materials.

The association between hukou type and GHQ-12 scores varied by the level of income, whereby migrants with a Shenzhen hukou scored lower on GHQ-12 than those with a non-Shenzhen hukou. The difference in GHQ-12 scores between hukou types was more noticeable for the middle- and high-income groups than for the low-income group. The relationship between hukou type and GHQ-12 also varied by physical health. Among those who rated their physical health as good, non-Shenzhen hukou holders had higher GHQ-12 scores than hukou holders; there was little difference for other physical health levels.

The association between income level and GHQ-12 showed different patterns across physical health levels. A sharp drop in GHQ-12 scores between the low- and the middle-income group was found for migrants who rated their physical health as fair or poor. The pronounced negative association between income and GHQ-12 scores were flattened for migrants who rated their physical health as better than fair/poor.

**Fig. 5:**
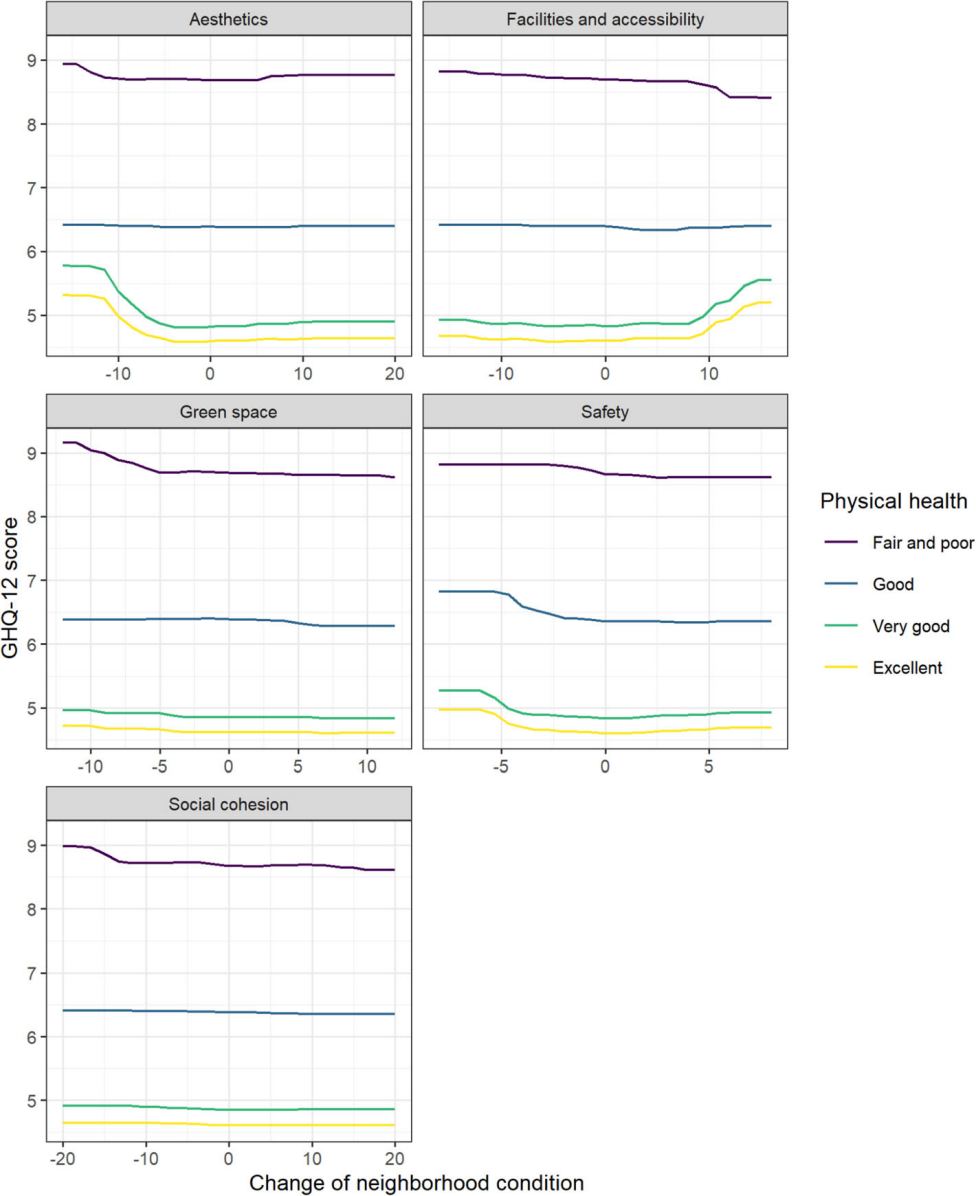
Bivariate partial dependence plots of physical health and neighborhood characteristics

Even though changes in neighborhood characteristics were not strongly related to migrants’ GHQ-12 scores (Fig. 1), the associations varied across different physical health levels. Figure 5 shows the associations between changes in perceived neighborhood characteristics and GHQ-12 scores for migrants with different physical health levels. Respondents who rated their physical health as very good or excellent showed a sharper increase in GHQ-12 scores when they experienced a decline in neighborhood aesthetics and safety post-migration. A similar trend appeared between changes in perceived safety and GHQ-12 for migrants with good physical health. Yet, for migrants with fair/poor physical health, their GHQ-12 scores remained high regardless of whether changes in aesthetics and safety were experienced. In addition, we observed a sharp increase in GHQ-12 scores when migrants with very good or excellent physical health experienced a substantial improvement (above 10) in neighborhood facilities and accessibility. As for changes in green space and social cohesion, those who rated their physical health as fair/poor showed a more pronounced negative association between neighborhood changes and GHQ-12 scores compared to other physical health groups.

## Discussion

We conducted a machine learning-based analysis to examine the associations between perceived changes in multiple neighborhood physical and social environmental characteristics pre- and post-migration and migrants’ mental health in Shenzhen, a metropolis whose population is largely composed of migrants. We also addressed how important changes in neighborhood characteristics are related to individual-level characteristics in an exploratory manner, including their level of interactions across variables—an issue that has rarely been examined.

### Experience of environmental neighborhood change

We found that, in general, migrants experienced a decline in perceived neighborhood aesthetics, safety, and social cohesion post-migration. This result supports studies that have reported that migrants are likely to end up in less desirable neighborhoods due to their disadvantaged position in the local housing market [55]. Unaffordable housing prices in Shenzhen [56], combined with a lack of family support, social capital, and financial capital, contributed to migrants’ higher level of housing stress and poorer neighborhood choice in Shenzhen [57]. Meanwhile, migrants’ social integration may negatively affect their perception of social aspects of the neighborhood environment [17], contributing to their reduced perceptions of neighborhood social cohesion in the host city.

In addition to the experienced decline in neighborhood aesthetics, safety, and social cohesion, our respondents also reported an improvement in neighborhood facilities and accessibility and in green space in Shenzhen. Since the majority of internal migration flows in China are rural to urban and/or from less developed cities to metropolitan areas [58], migrants may experience an overall improvement in urban infrastructure provision at their destination in Shenzhen. The increased green space perception in Shenzhen could be attributed to the city’s subtropical climate, which makes it greener than most other Chinese cities, especially northern cities: In 2018, the green coverage rate of Shenzhen’s built-up area was 45.1%, making it a top-ranking ecological environment among Chinese cities [59].

### Neighborhood change and mental health

Our results suggest that post-migration, migrants moved to neighborhoods perceived as less safe and had poorer mental health. However, an improvement in neighborhood safety was not associated with mental health benefits. Similarly, non-linear associations with mental health were also found for neighborhood aesthetic quality and green space, where only the association between a decline in neighborhood characteristics and poor mental health was identified. Empirical studies have suggested that neighborhood safety, aesthetic quality, and green space contribute to residents’ mental health [16, 60, 61]. Therefore, it is reasonable to conclude that a decline in such neighborhood characteristics may contribute to mental illness. Furthermore, due to institutional barriers caused by hukou, and a lack of social support in the host city, migrants struggle to maintain their neighborhood quality in the host city [62], which may induce additional stress as a result of ending up in deprived neighborhoods, putting them at risk for mental illness [17].

For neighborhood social cohesion, we found that a perceived decline in such cohesion is associated with poor mental health, and that a perceived improvement is associated with better mental health. Previous research has shown positive effects of perceived neighborhood social cohesion in terms of improving residents’ life satisfaction [63] and enhancing their social contact and support [64], which contribute to good mental health.

In contrast to prior studies [60, 65] we found that people who moved to neighborhoods with better facilities and accessibility post-migration reported poorer mental health. However, some studies did find an association, albeit an insignificant one, between mental health and access to neighborhood facilities and services [66–68]. Similarly, a UK panel data-based study [69] on internal migrants found that moving to a less deprived physical environment was associated with poor mental health. In fact, most evidence on the health-promoting role of neighborhood facilities and accessibilities concerns residents’ physical (rather than mental) health, which is promoted by encouraging physical activities and more active traveling modes, such as walking and cycling [70–72]. Considering the limited and mixed results linking neighborhood facilities and accessibilities to mental health, we speculate that while better neighborhood facilities and accessibility may indirectly contribute to residents’ mental health by improving their physical health, there are potential stressors that may offset the effect. For instance, neighborhoods with better facilities and accessibility may attract more visitors, increasing the density and crowdedness of the neighborhood, which will have negative impacts on mental health [73]. In sum, our results suggest that neighborhood changes and mental health have a complex, non-linear relationship. The mechanisms behind the patterns require more in-depth and context-specific investigations.

In line with an earlier study concerning the general population [12], the variable importance showed that, in general, neighborhood characteristics were less important in explaining migrants’ mental health outcomes compared to their individual-level characteristics (e.g., physical health, income, and hukou). A reason for this could be that individual-level characteristics, such as income and hukou, may not only serve as influential factors for migrants’ mental health [10, 74], but also enable or constrain people’s selection of neighborhood types [75]. Among the perceived neighborhood characteristics, it turned out that neighborhood safety in the host city was essential. This result is congruent with earlier work that reported that safety in the living environment is related more strongly to migrants’ reduced psychological stress level in the host city than any other neighborhood physical characteristic [76].

### The importance of physical health

Physical health is often strongly related to migrants’ mental health [77], as it is for general populations [10]. Poor physical health may limit people’s physical activity [77], which increases the risk for mental illness [78]. In addition, in our models we found striking interactions between self-rated physical health and income, hukou, and three neighborhood characteristics (green space, aesthetics, and safety). For instance, people with poor physical health were at greater risk for mental illness if they had a low personal income compared to those with better physical health. Similarly, the relationships between changed neighborhood-level characteristics and mental health varied across physical health groups. Specifically, migrants with fair/poor physical health were more sensitive to a decrease in perceived neighborhood green space.

Our results also showed that poorer mental health was associated with worsening perceived neighborhood aesthetics and safety only for those with at least very good physical health. Such differences across physical health groups may be due to the fact that physical health status may influence people’s perception of their neighborhood environment [79]. Physical health conditions were also found to be related to the way and frequency with which people interact with their neighborhood environment [80]. For example, physically healthier people tend to go out more frequently than less healthy people, and thus their mental health response could be more sensitive to a decline in neighborhood aesthetics and safety.

### Other individual-level characteristics

Income and hukou type were also found to be important for migrants’ mental health in the host city. Several studies have suggested an income–health gradient, whereby a high personal income is associated with better mental health [81, 82]. A positive association between income, life satisfaction, and mental health may be especially important for migrants, because they mainly move to cities to earn higher incomes [6]. In our study, hukou was also of central importance for migrants’ mental health, as highlighted in other Chinese studies [3, 83]. Hukou is strongly related to social welfare, health services, and the provision of other public services [84]. The difference between local hukou and non-local hukou holders regarding health insurance and social welfare indicates great health inequality in cities like Shenzhen [85]. Migrants frequently suffer from such inequality due to difficulties in transferring their hukous to the host city [10].

### Strengths and limitations

Our study had numerous strengths. First, we focused on neighborhood changes pre- and post-migration, something that has rarely been done. Second, we incorporated multiple neighborhood characteristics rather than focus on a single one. This approach took into account that different neighborhood characteristics are likely to co-vary and influence each other. Third, in order to be methodologically innovative, we employed a data-driven random forest to assess the variable importance of neighborhood characteristics and their interactions, while going beyond linear relations.

Our study also had a number of limitations. First, the cross-sectional nature of the research design did not allow for causal statements and reverse causality remains an issue. We cannot preclude that people with poor mental health tend to have a more negative perception of their residential neighborhood and that those people self-select into more deprived residential areas. Second, although the perceived neighborhood characteristics have been found to be more influential for mental health than objective measures [86], we cannot rule out that people’s perceptions vary, so their retrospective neighborhood assessments might have been prone to recall bias, which can be potentially influenced by various factors such as respondents’ sociodemographics and the length of residence in Shenzhen. Finally, due to a lack of data on migrants’ mental health conditions prior to moving to Shenzhen, we were not able to realize longitudinal analyses. Given these limitations, we encourage future studies to employ longitudinal designs that incorporate both objective and perceived neighborhood characteristics, while measuring changes in mental health over time.

## Conclusions

This study was among the first to examine recalled changes in the perceived physical and social residential environments on internal migrants’ mental health. We found that perceived negative changes of neighborhood aesthetics, safety, green space, and social cohesion were associated with poor mental health in the post-migration stage for migrants in Shenzhen. Yet, the associations between neighborhood environment changes and migrants’ mental health were minor. Three individual-level characteristics—namely physical health, income, and hukou—were among the most important factors associated with migrants’ mental health. In addition, physical health interacted strongly with other variables (e.g., income and hukou) when correlating with mental health.

## Supplementary Information

**Additional file 1: Figure S1** Bivariate partial dependence plots of physical health and age. **Figure S2** Bivariate partial dependence plots of physical health and education. **Figure S3** Bivariate partial dependence plots of education and age. **Figure S4** Bivariate partial dependence plots of Hukou type and age. **Figure S5** Bivariate partial dependence plots of income group and age. **Figure S6** Bivariate partial dependence plots of migration context and age. **Figure S7** Bivariate partial dependence plots of sex and education. **Figure S8** Bivariate partial dependence plots of employment and education. **Figure S9** Bivariate partial dependence plots of hukou type and education. **Figure S10** Bivariate partial dependence plots of migration context and education. **Figure S11** Bivariate partial dependence plots of hukou type and employment. **Figure S12** Bivariate partial dependence plots of migration context and education. **Figure S13** Bivariate partial dependence plots of sex and employment. **Figure S14** Bivariate partial dependence plots of hukou type and sex.

## Data Availability

The data that support the findings of this study are available on request from the corresponding author [MY]. The data are not publicly available because they contain information that could compromise research participants’ privacy/consent.

## Abbreviation

GHQ-12: The 12-item General Health Questionnaire
