# Supplementary material for: Assessing the perceived changes in neighborhood physical and social environments and how they are associated with Chinese internal migrants’ mental health
Source: BMC Public Health. 2021 Jun 28;21:1240. doi: 10.1186/s12889-021-11289-4 (PMC8240258; doi:10.1186/s12889-021-11289-4)
Supplement: Supplementary file 1 — Additional file 1: Figure S1 Bivariate partial dependence plots of physical health and age. Figure S2 Bivariate partial dependence plots of physical health and education. Figure S3 Bivariate partial dependence plots of education and age. Figure S4 Bivariate partial dependence plots of Hukou type and age. Figure S5 Bivariate partial dependence plots of income group and age. Figure S6 Bivariate partial dependence plots of migration context and age. Figure S7 Bivariate partial dependence plots of sex and education. Figure S8 Bivariate partial dependence plots of employment and education. Figure S9 Bivariate partial dependence plots of hukou type and education. Figure S10 Bivariate partial dependence plots of migration context and education. Figure S11 Bivariate partial dependence plots of hukou type and employment. Figure S12 Bivariate partial dependence plots of migration context and education. Figure S13 Bivariate partial dependence plots of sex and employment. Figure S14 Bivariate partial dependence plots of hukou type and sex. [file 12889_2021_11289_MOESM1_ESM.pdf]

Supplementary materials: bivariate interactions between individual-level characteristics and mental health (GHQ)

Figure S 1 Bivariate partial dependence plots of physical health and age

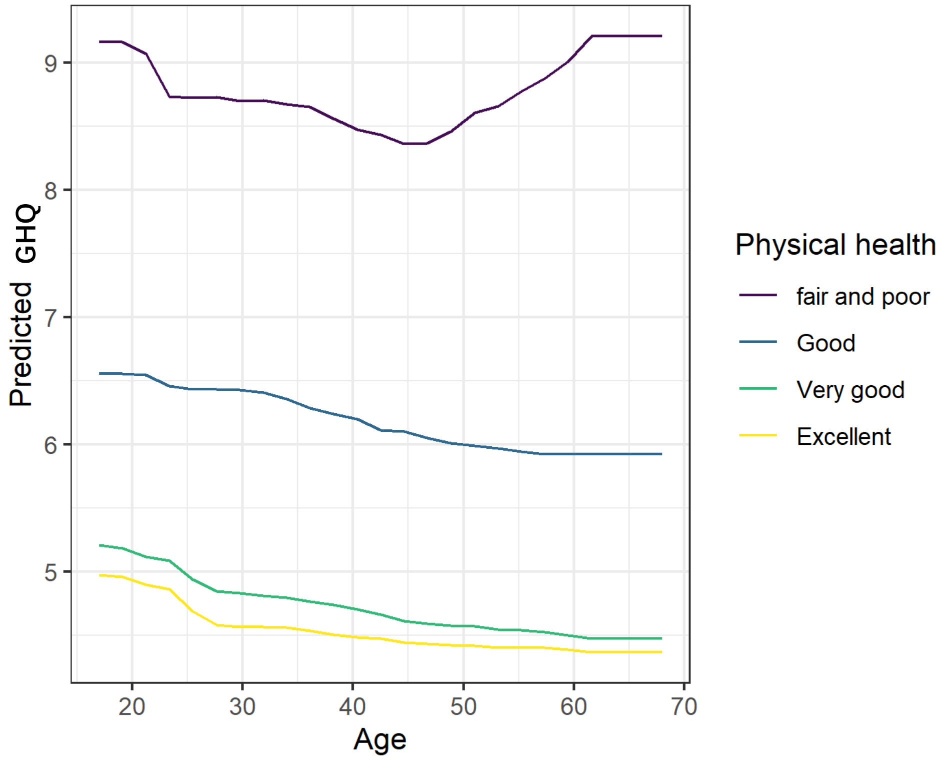

Figure S 2 Bivariate partial dependence plots of physical health and education

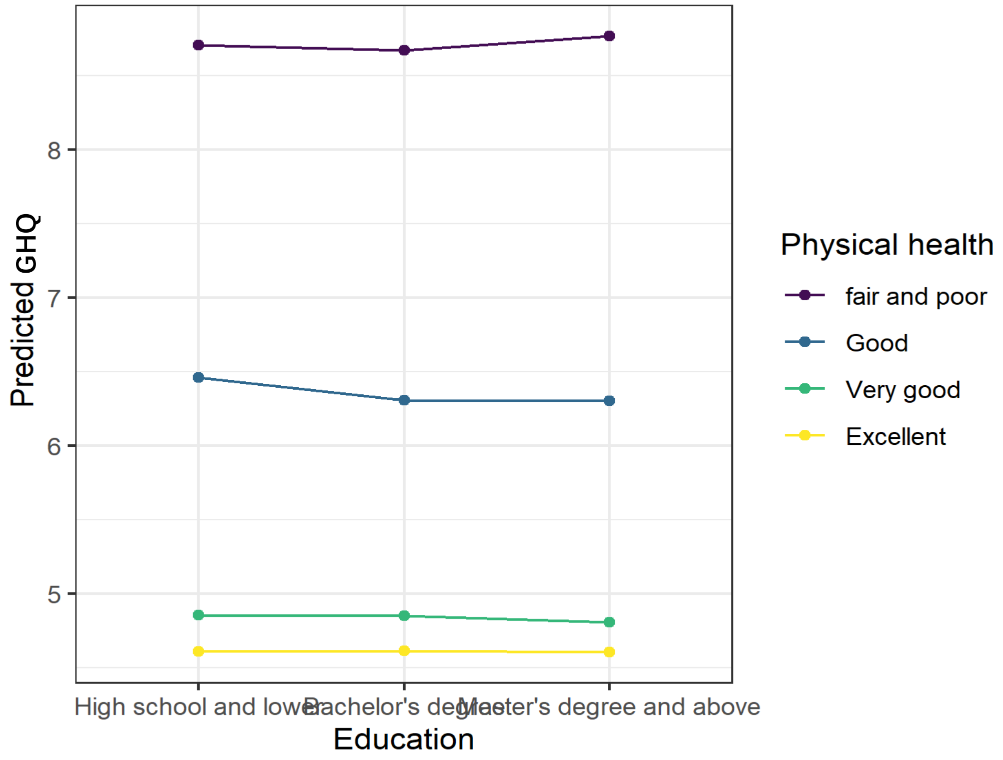

Figure S 3 Bivariate partial dependence plots of education and age

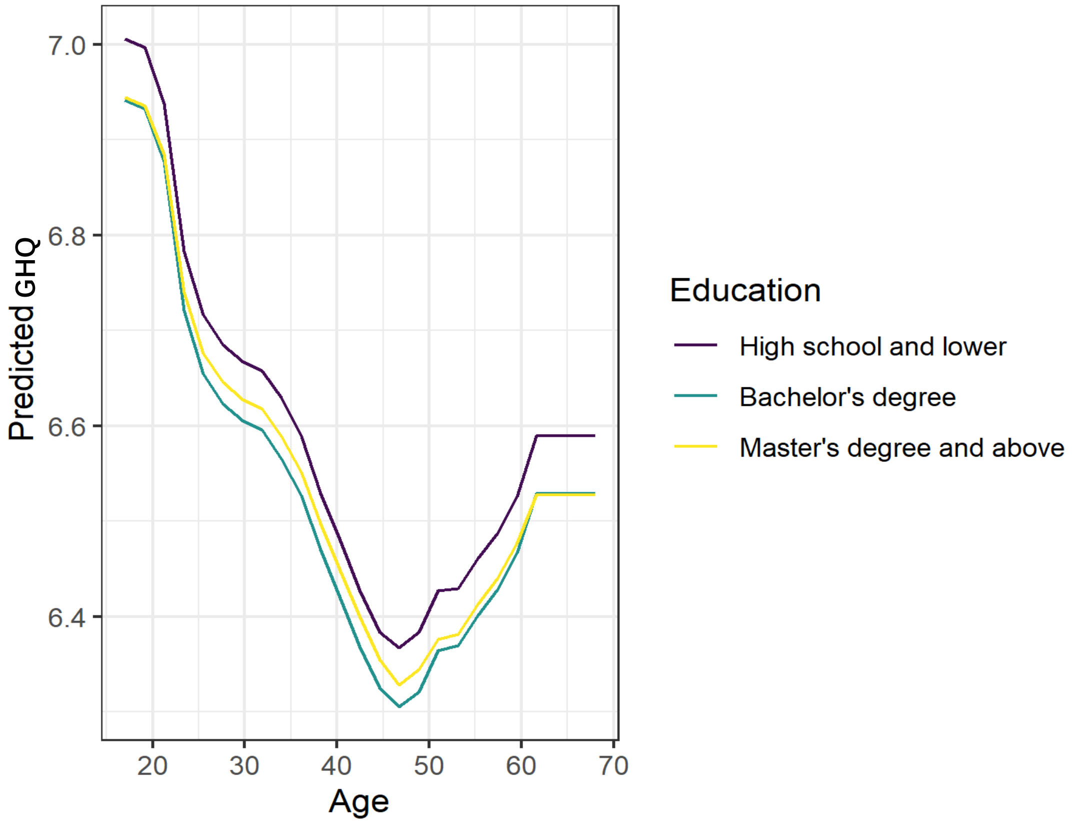

Figure S 4 Bivariate partial dependence plots of Hukou type and age

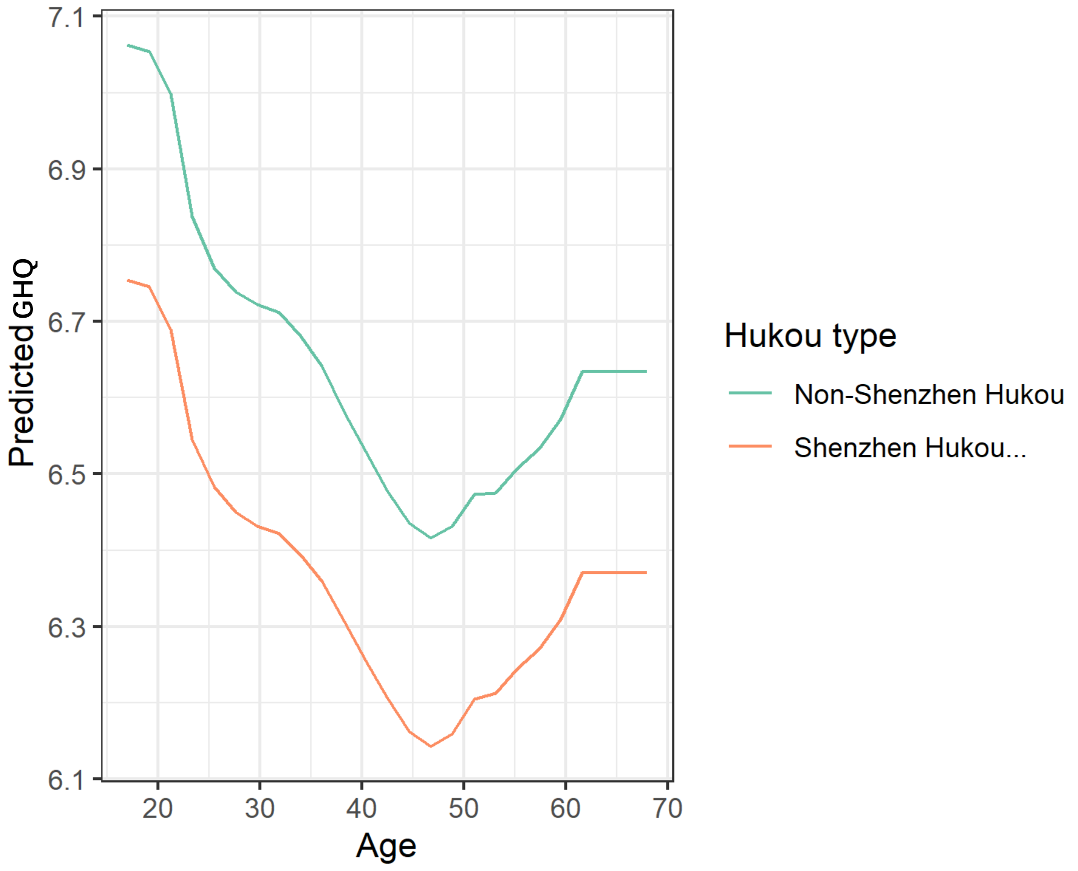

Figure S 5 Bivariate partial dependence plots of income group and age

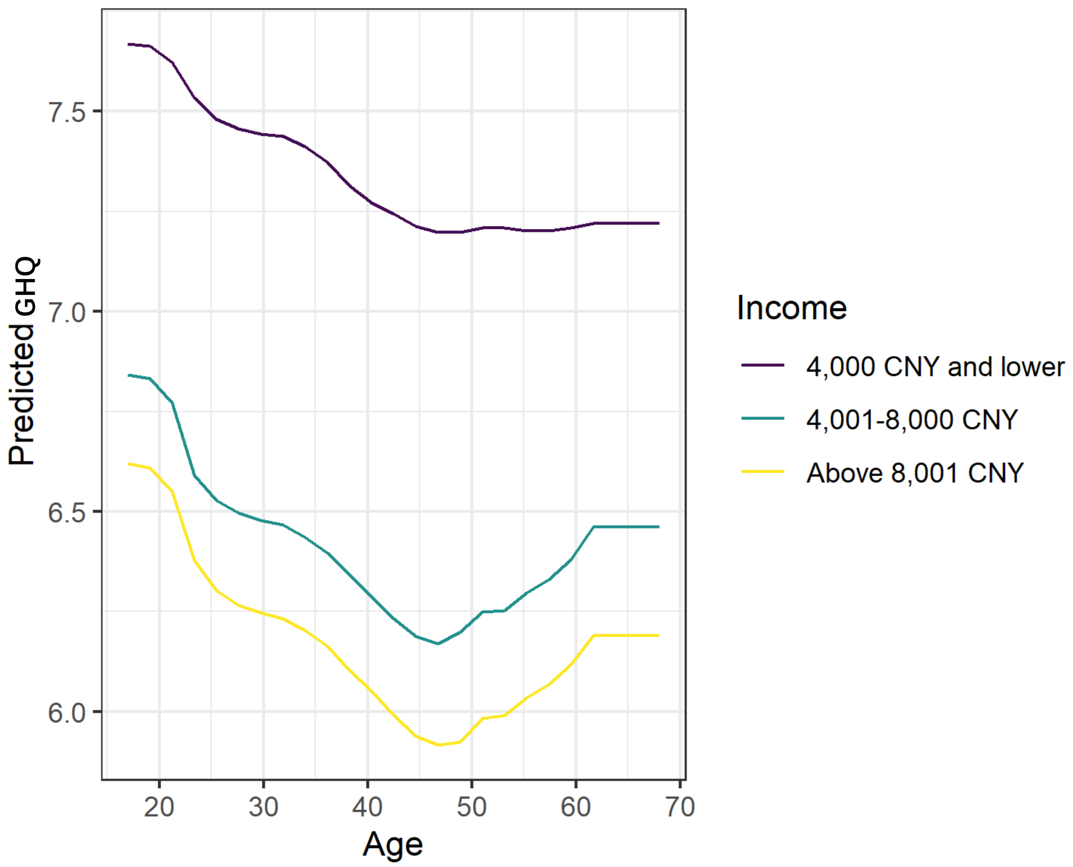

Figure S 6 Bivariate partial dependence plots of migration context and age

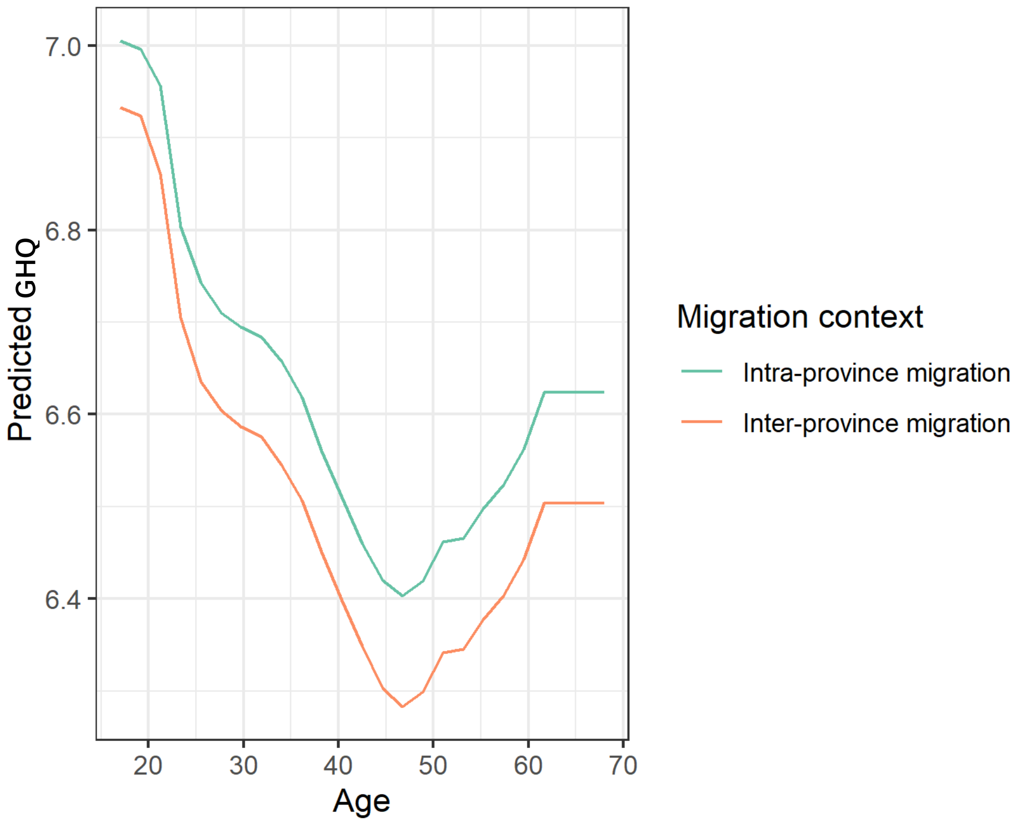

Figure S 7 Bivariate partial dependence plots of sex and education

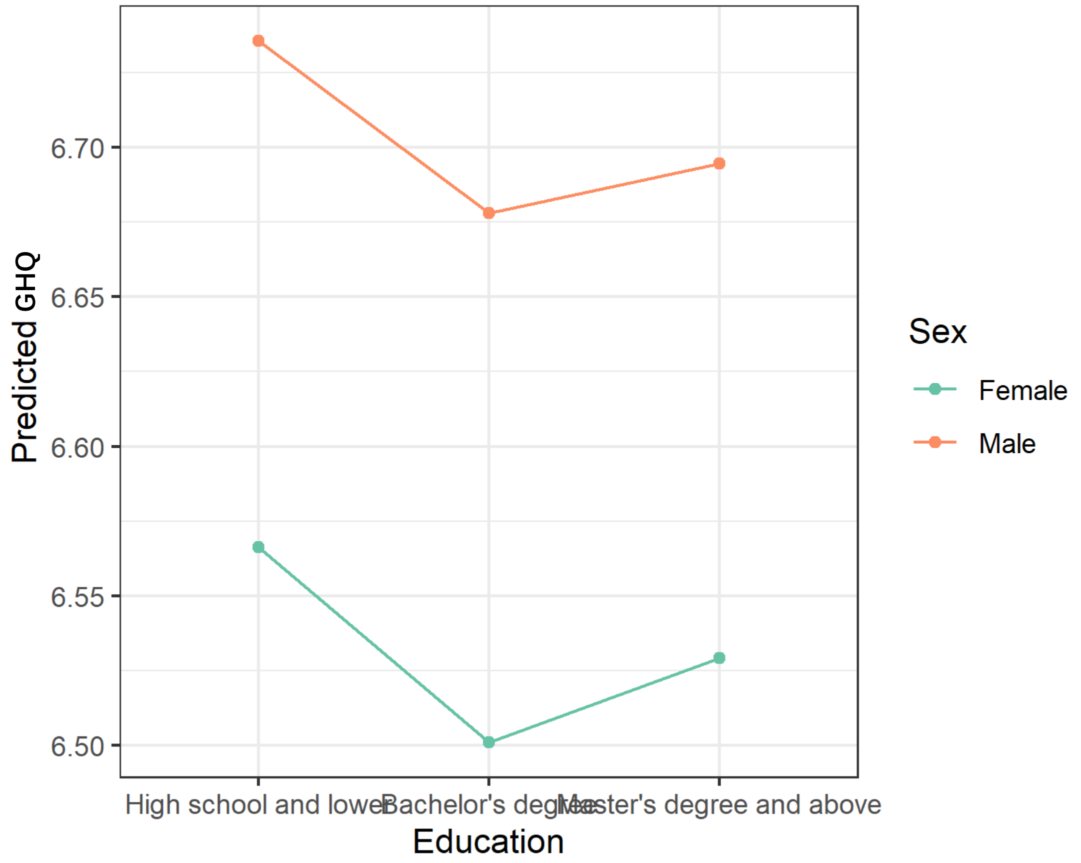

Figure S 8 Bivariate partial dependence plots of employment and education

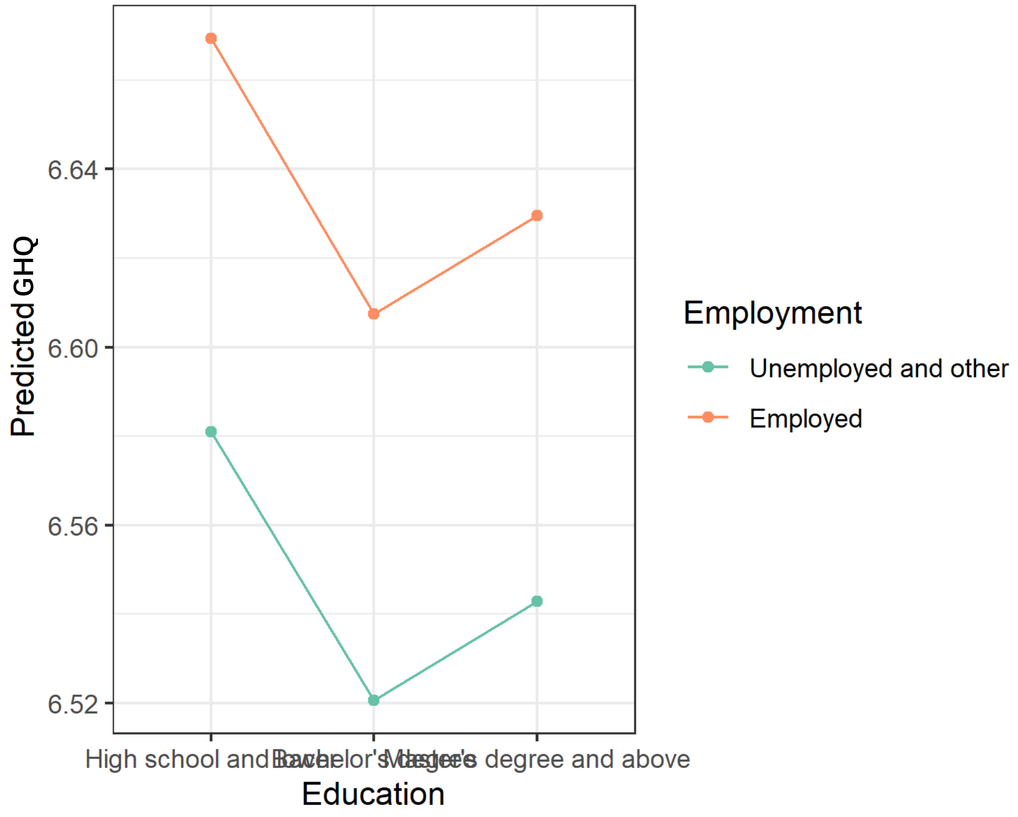

Figure S 9 Bivariate partial dependence plots of hukou type and education

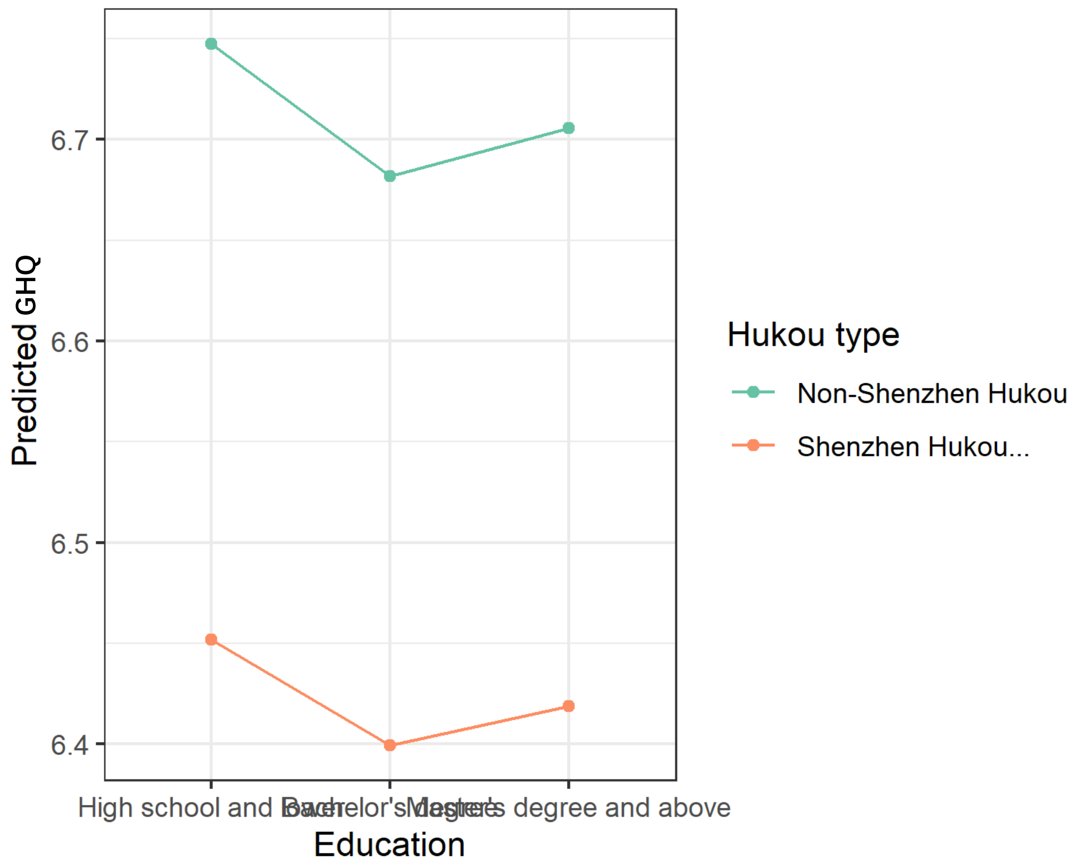

Figure S 10 Bivariate partial dependence plots of migration context and education

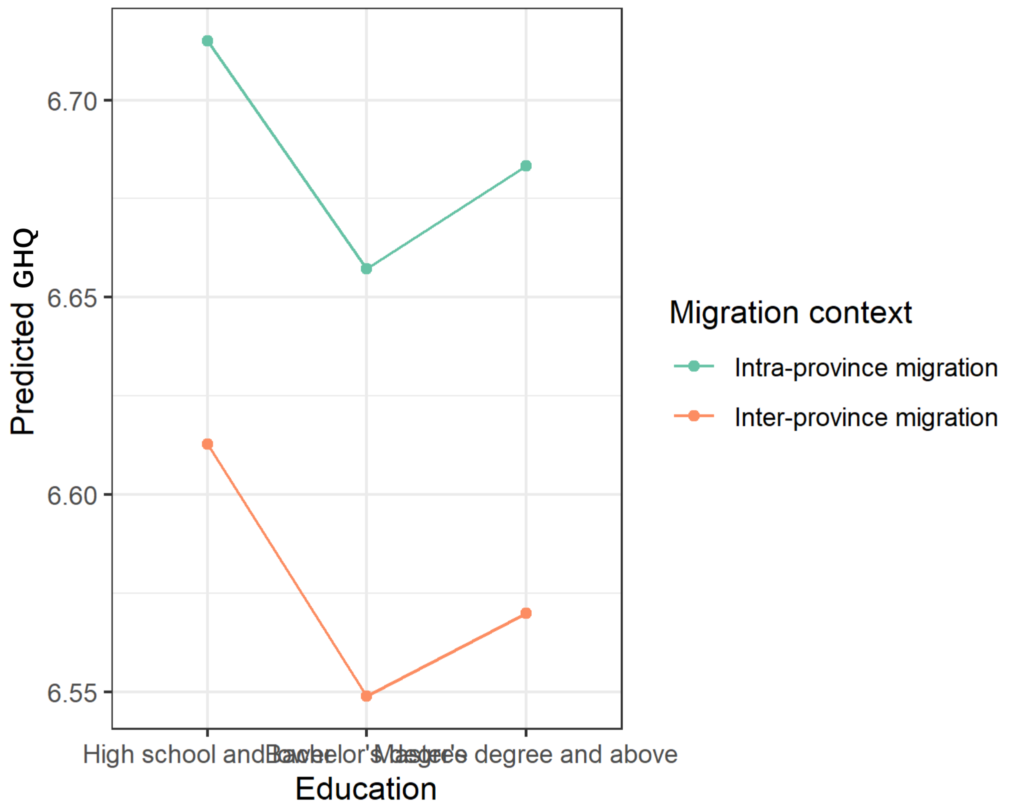

Figure S 11 Bivariate partial dependence plots of hukou type and employment

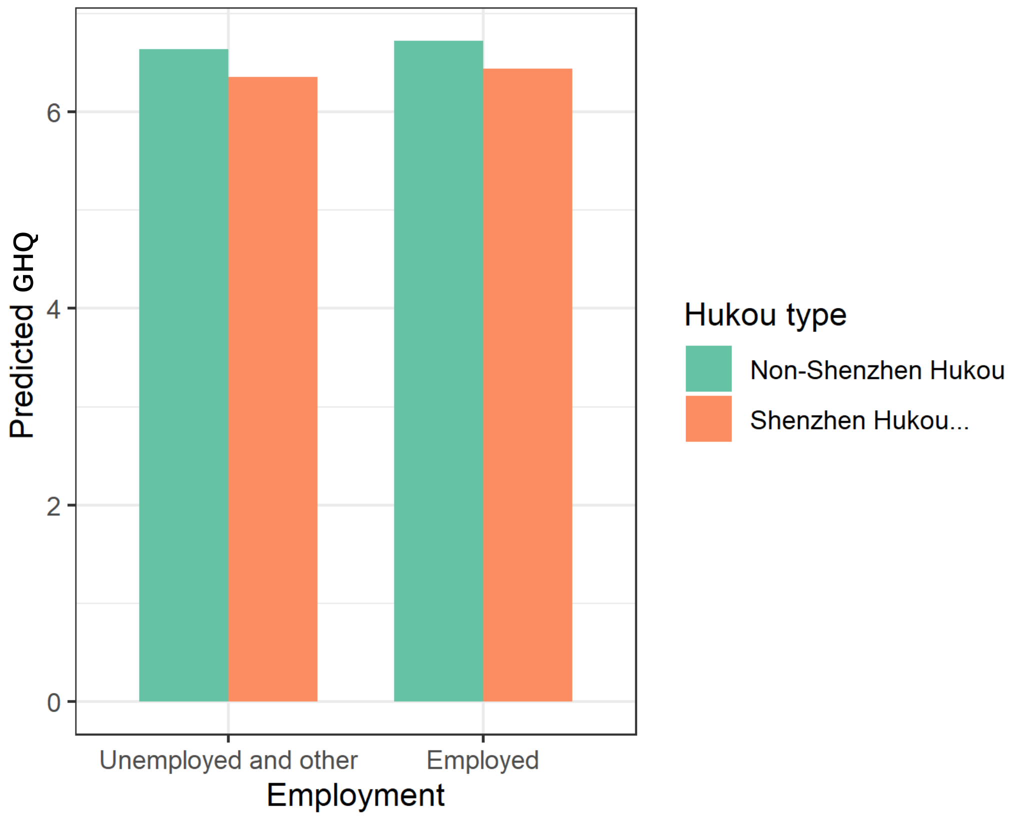

Figure S 12 Bivariate partial dependence plots of migration context and education

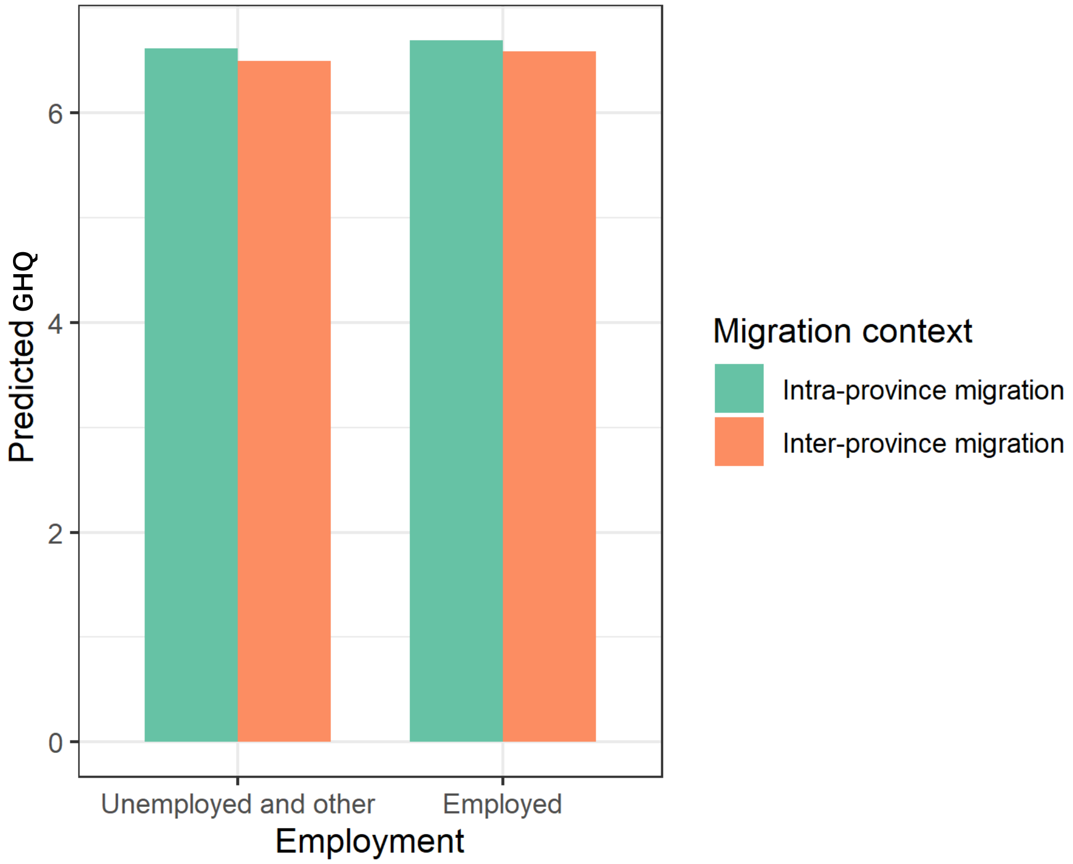

Figure S 13 Bivariate partial dependence plots of sex and employment

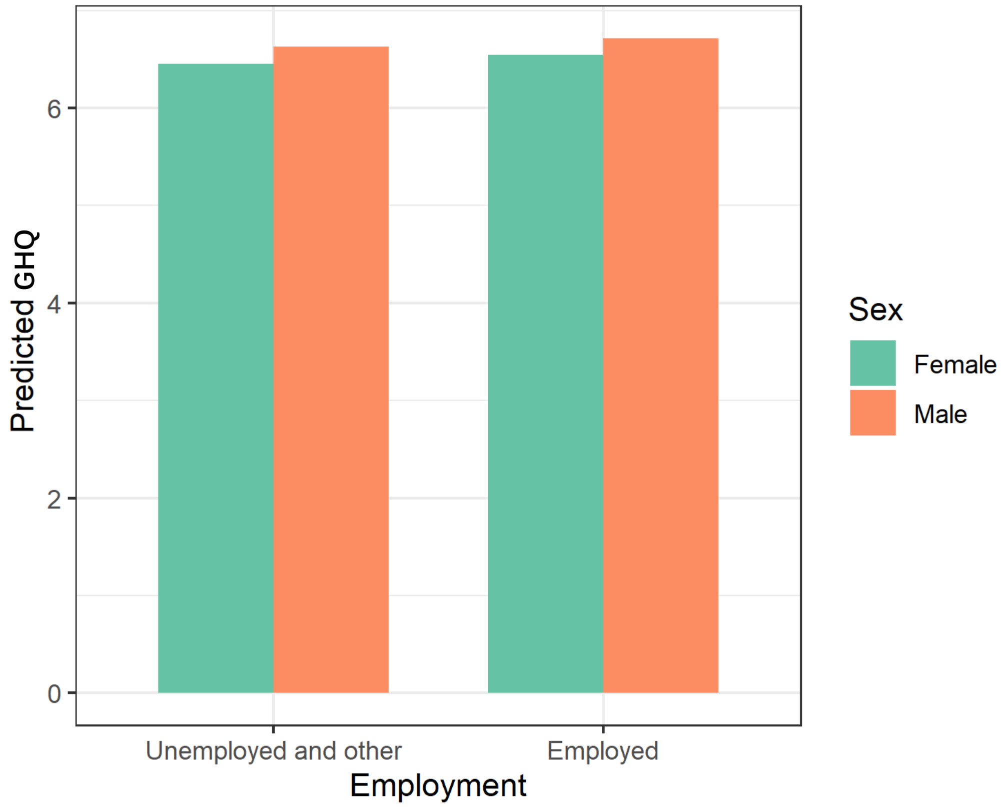

Figure S 14 Bivariate partial dependence plots of hukou type and sex

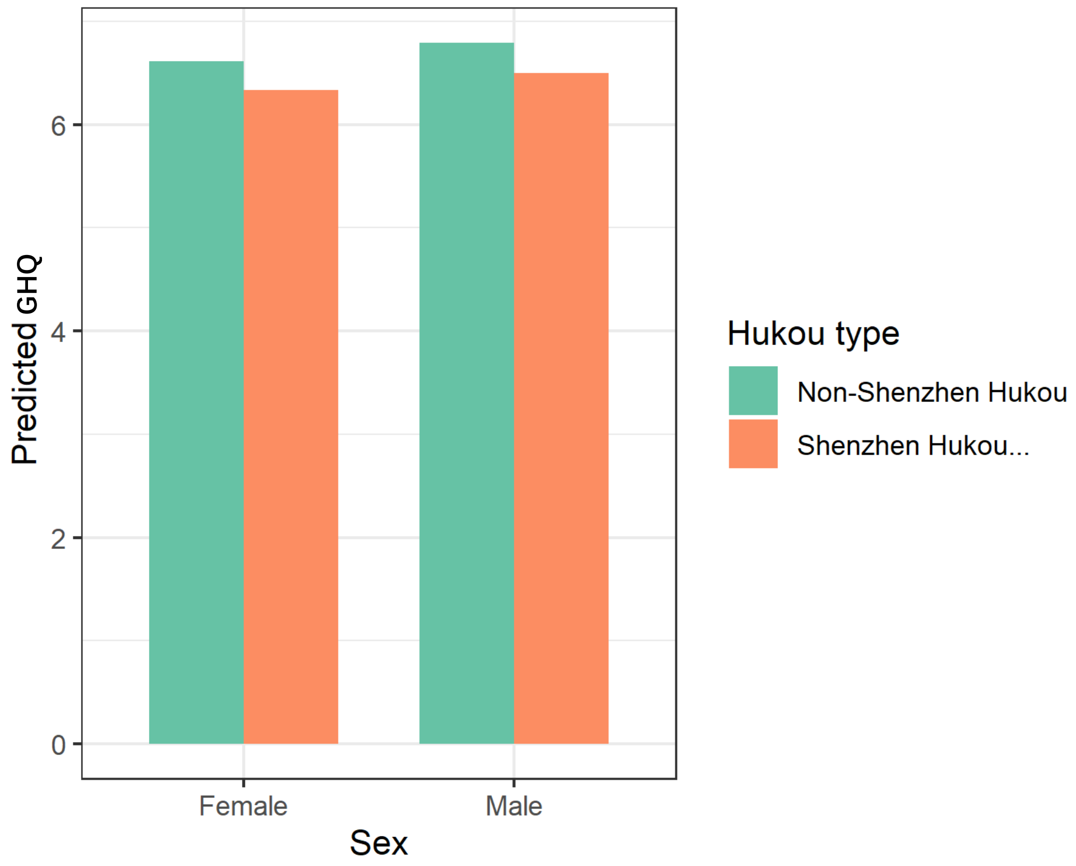

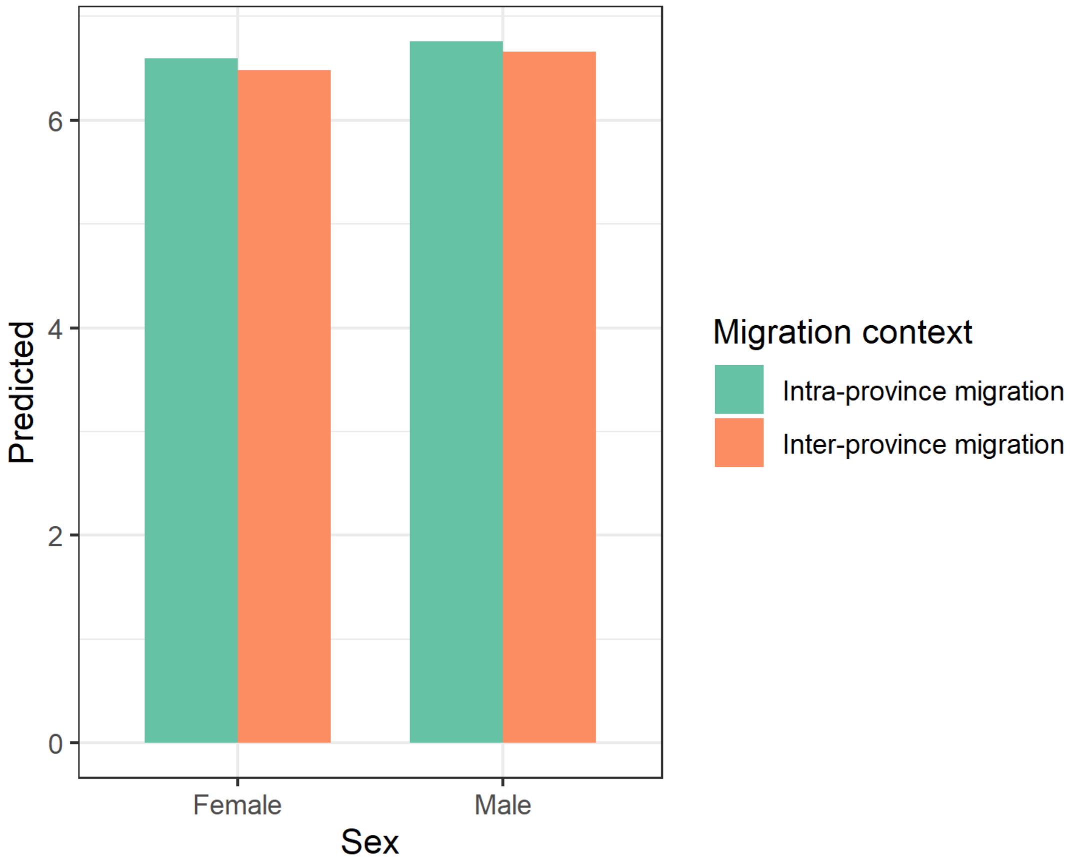
Predicted
0
2
4
6
Female
Male
Sex
Migration context
Intra-province migration
Inter-province migration
